# Diseases of the Heart and Circulation

**Published:** 1902-12-20

**Authors:** 


### Diseases of the Heart and Circulation.

**State of Heart after Death from Asphyxia.**—Some hours after death from asphyxia it is found that the right side of the heart and great veins opening into it are largely distended with blood, while the left side of the heart is empty or contains little blood. The cause of this has been ascribed to various influences. MacWilliam [1] has made experiments to find out what takes place from the moment of death onwards. The whole heart stops distended with blood, both auricles and both ventricles. This is brought about by the inability of the left ventricle to empty itself against the increased peripheral resistance caused by the contracted state of the small arteries. The right side of the heart becomes distended owing to the inability of the left side to pump on its blood efficiently. The pressure on the right and left ventricle in this condition is approximately the same, though the right ventricle contains a little more blood than the left, owing to its greater distensibility. The characteristic asphyxial condition of the heart as seen some hours after death is a post-mortem development due to rigor mortis in the cardiac muscle. The force of this in the left ventricle (as determined by a manometer) is about 30 mm. Hg. in the cat. The expulsive power of this is much more than would be necessary to expel its contents against the very low resistance present in the aorta after death. If the heart be watched after death it can be seen to contract as it passes into a state of rigor mortis. After asphyxia the right side of the heart dies slowly and gradually, much later than the left side. This has very little expulsive power, owing to its lateness in development, its gradual onset, and unequal distribution. Consequently the right side of the heart and great veins opening into it remain distended, as at the moment of death. Variations in the time of onset and strength of rigor influence the occurrence of these changes. If rigor is early and strongly marked, both ventricles may empty themselves; if it is slowly developed and feeble the left ventricle, as well as the right, remains distended. In chloroform poisoning, if the respiration has stopped before the heart beat, the heart usually stops beating, distended in all its parts. And if the heart stops first, it is found largely distended. In both cases the subsequent rigor empties the left side. Hence post-mortem evidence gives no indication whether death has been initiated by cardiac or by respiratory failure. After death from hæmorrhage both ventricles go into marked rigor, and are found contracted and empty; the left auricle is small, whilst the right auricle contains a fair amount of blood.

**Venous Thrombosis in Heart Disease.**—Two cases of this rare complication of heart disease have been recorded by Hale White,[2] with an interesting discussion of the subject. The two acute diseases which lead to thrombosis are typhoid fever and influenza; and the two chronic diseases in which it occurs are tubercle and cancer; in all four of which there is no particular difficulty in the circulation. On the other hand thrombosis is very rare in heart disease even with considerable circulatory failure. It is therefore a condition which is not due to any single factor, certainly not to retardation of the circulation. The thrombosis that occurs in heart disease is nearly always in the veins of the arms, head and neck; in curious distinction to the venous thrombosis of typhoid fever, influenza, tubercle and cancer, in which it is nearly always in the legs. The venous thrombosis of heart disease is generally in young people, more often in women. The affection of the heart is mitral disease, with or without aortic valve disease; and in many cases there is also an adherent pericardium. The thrombosis appears during the period of extreme failure of the heart. It takes place in the internal jugulars, the innominate veins, or the lower parts of the subclavian or external jugulars, especially on the left side, and spreads backwards. The walls of the veins themselves are not diseased. The thrombosed veins are tender and firm to the touch. The symptoms are a quickly appearing lividity and œdema affecting the face, neck, and upper extremities, and extending about two inches on the thoracic wall. It is a very serious symptom and generally has a fatal termination. The cause of the thrombosis is unknown, but the determining factor is probably the presence of bacteria.

**Persistent Hereditary Œdema of the Lower Limbs.**—Rolleston [3] has described the occurrence of a peculiar form of œdema which affected a woman and two out of her seven children. The œdema was only troublesome from the weight and size of the swollen legs, and did not cause any other bad results. It varied from time to time, gradually increasing when the patients were up, slowly receding when they were kept warm in bed. The skin was free from any structural change, and was natural in colour, but the circulation was feeble, pressure on the skin leaving an anæmic area for a much longer time than in normal persons. No recognised cause for the anæmia was discovered on careful examination. In the mother's case the condition developed at the age of 10 years, in that of the children at the ages of 10 and 13 years respectively. The only cases hitherto described resembling the above are those published by Milroy, in which out of 97 individuals in six generations, 22 had solid œdema of one or both legs without any special inconvenience or progressive increase in the disease; the condition was congenital except in one case in which it developed at the age of 12 years. Milroy's cases were thus not only hereditary, but also (except one) congenital, whereas Rolleston's were only hereditary. The cause of this peculiar disease is very doubtful—possibly it depends on some inherent defect in the small blood-vessels, which allows excessive transudation to occur on very slight provocation.

**Aneurysm of the Aorta.**—Dickinson[4] has published four cases of aneurysm of the aorta in which the cause was apparently hypoplasia of its wall. The cases were either saccular or fusiform, and each of them showed not the slightest tendency to cure, and ended in rupture. It is easy to see that congenital delicacy of the arteries when it exists must predispose to the formation, and favour the development, of aneurysm. In this connection it is worth notice that aneurysms in women, as a rule, go from bad to worse more rapidly than in men, and are much more prone to end in rupture. External rupture in particular is nearly as common in women as in men notwithstanding the relative great rarity of aneurysm. Now women are notoriously more liable to hypoplasia than men, and the suggestion presents itself that some degree of hypoplasia, not necessarily to be seen unless looked for, may be the reason for the more untoward course of their aneurysms. And so it may be with children and young persons when they suffer from aneurysm. Again, arterial hypoplasia is one of the leading pathological facts of hœmophilia—which suggests that the prospect of cure by deposition of fibrin is not good when the cause of an aneurysm is hypoplasia, even though no true hœmophilia is present.

[1] Brit. Med. Jour., April 5. [2] Clinical Jour, Aug. 27. [3] Lancet, Sept. 20. [4] Lancet, Aug. 9.

*(To be continued.)*

